# Directly Transforming PCR-Amplified DNA Fragments into Plant Cells Is a Versatile System That Facilitates the Transient Expression Assay

**DOI:** 10.1371/journal.pone.0057171

**Published:** 2013-02-26

**Authors:** Yuming Lu, Xi Chen, Yuxuan Wu, Yanping Wang, Yuqing He, Yan Wu

**Affiliations:** State Key Laboratory of Hybrid Rice, Department of Cell and Developmental Biology, College of Life Sciences, Wuhan University, Wuhan, People’s Republic of China; RIKEN Plant Science Center, Japan

## Abstract

A circular plasmid containing a gene coding sequence has been broadly used for studying gene regulation in cells. However, to accommodate a quick screen plasmid construction and preparation can be time consuming. Here we report a PCR amplified dsDNA fragments (PCR-fragments) based transient expression system (PCR-TES) for suiting in the study of gene regulation in plant cells. Instead of transforming plasmids into plant cells, transient expression of PCR-fragments can be applicable. The transformation efficiency and expression property of PCR-fragments are comparable to transformation using plasmids. We analyzed the transformation efficiency in PCR-TES at transcription and protein levels. Our results indicate that the PCR-TES is as versatile as the conventional transformation system using plasmid DNA. Through reconstituting *PYR1*-mediated ABA signaling pathway in *Arabidopsis* mesophyll protoplasts, we were not only validating the practicality of PCR-TES but also screening potential candidates of CDPK family members which might be involved in the ABA signaling. Moreover, we determined that phosphorylation of ABF2 by CPK4 could be mediated by ABA-induced PYR1 and ABI1, demonstrating a crucial role of CDPKs in the ABA signaling. In summary, PCR-TES can be applicable to facilitate analyzing gene regulation and for the screen of putative regulatory molecules at the high throughput level in plant cells.

## Introduction

Transient expression system is an important research approach for conducting cell-based assays in plant and animal cells. In comparison with stable transformation system a transient expression assay is of quick analyzing advantage, which may not interfere with the stability of host genome [1], [2]; therefore, it is widely used for studying transient activities of genes in cells [3]–[6].

In order to analyze the function of a gene in plant cells, a number of strategies of transient expressions are commonly used in laboratories. For instance, microinjection enables delivery of molecules into single cells with a set of microinjector [7]. Biolistic bombardment allows delivery of foreign DNA into cells to achieve transient and stable transformations [8], [9]. *Agrobacteria* mediated transformation method has been applied for introducing plasmid DNA into plant cells, thus, stable transgenic plants can be generated [5], [10]. In addition, the polyethylene glycol (PEG) mediated transformation serves as an efficient system for analyzing gene regulation at the single cell level [6], [11]–[13]. For example, using the mesophyll protoplasts transfection approach, the reconstitution of ABA receptor PYR/RCAR dependent signaling pathway is determined [11]. In response to ABA, PYR/RCAR recruits ABI1 (phosphotase 2C), resulting in the activation of SnRK2.6 kinase and ABA responsive transcription factors including ABF2 in plant cells [11]. The interaction between CPK23 (one of calcium-dependent protein kinases, CDPKs) and SLAC1 (a slow-anion channel) is also demonstrated in the transient expression assay with mesophyll protoplasts [13]. Moreover, the importance of CDPKs’ activities in the innate immune signaling pathways has also been concluded with transient expression assay in protoplasts of *Arabidopsis* [12].

No matter which kind of transient expression strategies one would choose, the traditional fashion is designated to transform plasmid DNA into cells. Thus, constructing the gene of interest into a plasmid and purifying the prepared plasmid DNA become the must-have procedure. In order to assess the transient expression of a gene in protoplasts, enough amount of high quality plasmid DNA is required [6]. However, several exceptions which were demonstrated to use PCR amplified double stranded DNA (dsDNA) fragments (PCR-fragments) for the transient expression assay in mammalian cells [14], [15] are noted. Transformation with PCR-fragments composing of CAT (Chloramphenicol Acetyltransferase) reporter gene and bacteriophage T7 promoter sequences into *293-T7* cells was reported [14]. In a high throughput transient expression assay, the paramagnetic beads coated with PCR-fragments are reportedly delivered into various mammalian cell lines [15]. These reports provide evidences to the fact that PCR-fragments may be applicable for studying the gene function in mammalian cells. However, transformation of PCR-fragments into plant cells remains unclear.

Here, we established a transformation system with PCR-fragments named PCR-TES (PCR-fragment based Transient Expression System). Our results demonstrated that PCR-TES is as vital as the plasmid transformation system, which was tested in PEG-mediated protoplasts transformation as well as in biolistic bombardment transformation with leave tissues. Transformation of PCR-fragments can be time-saving and efficient for the preliminary evaluation of a gene function. Based on PCR-TES, we screened 24 members of CDPK family, and determined that *CPK4* may play as a putative component in Ca^2+^-dependent ABA signaling pathway in *Arabidopsis*.

## Results

### Transient Expression of PCR-fragments in Epidermal Cells

**Figure 1:**
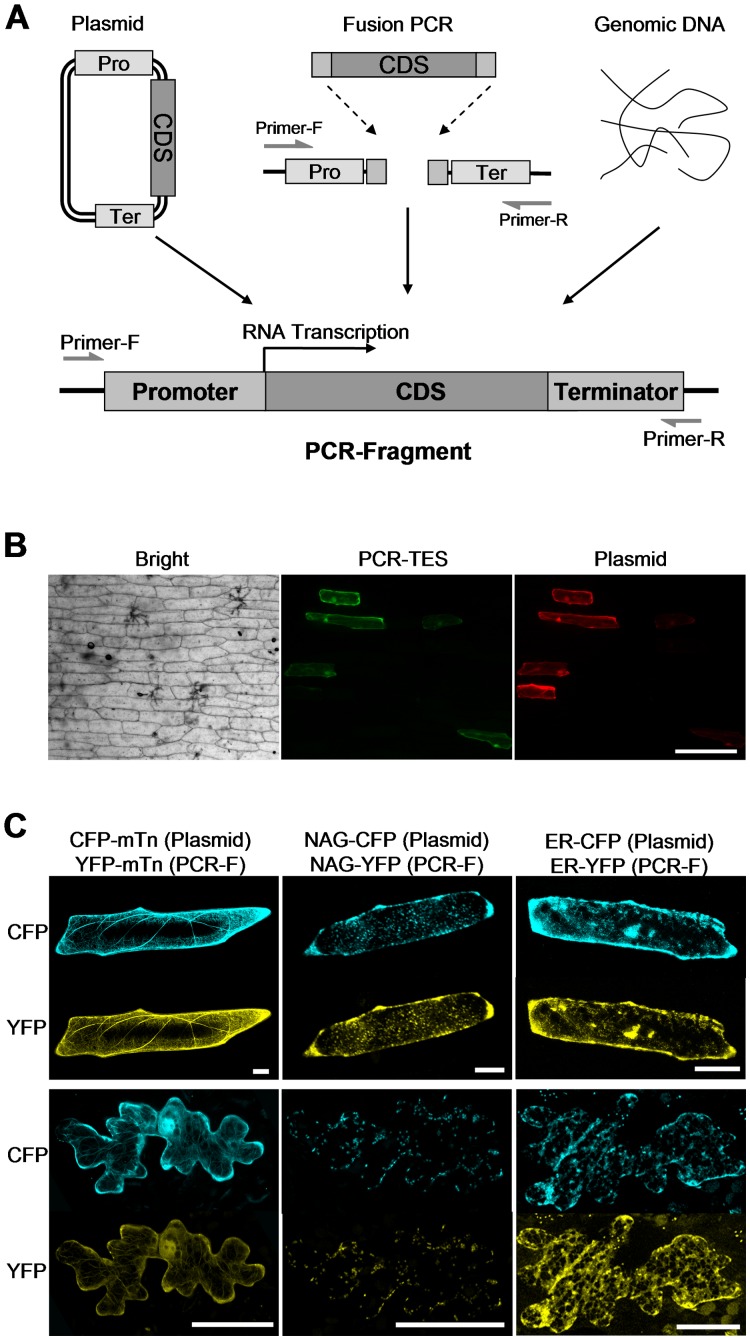
Transient expression of PCR-fragments in epidermal cells. (A) Schematic diagram to show the components in a PCR-fragment (not in scale) produced using a template plasmid (left), a source of genomic DNA (right), or fusion PCR (middle). (B) Expressing PCR-fragments in epidermal cells of *Onion* peels. Transformation with PCR-fragments *35S-GFP-NOS* (PCR-TES) and transformation with *p35S-Cherry* (Plasmid) were compared. Images were acquired after overnight incubation. Scale bar, 200 µm. (C) Transformations of PCR-fragments were examined in epidermal cells of *Onion* peels (upper panel) and *Arabidopsis* leaves (lower panel). PCR-fragments (PCR-F) of *YFP-mTn*, *NAG-YFP* and *ER-YFP* were cotransformed with their correspondent plasmids (Plasmid) *p35S-CFP-mTn*, *p35S-NAG-CFP* and *p35S-ER-CFP* through bombardment, respectively. Scale bars, 40 µm.

The components designed in the cassette of a PCR-fragment include a promoter, such as CaMV 35S promoter [16] or UBQ10 promoter [17], coding sequences of the gene of interest (CDS), and the terminator (NOS) (Figure 1A). Initially, PCR-fragments were generated through PCR amplification from the template plasmid DNA which contains the backbone of vector *pUC18*. The pair of universal primers PVU-F (located at 180 bp upstream of the promoter) and PVU-R (located at 100 bp downstream of NOS terminator) was used (Figure 1A). Amplified PCR-fragments were briefly purified with the extraction of phenol/chloroform and precipitated in ethanol [18]. In the first task, we prepared PCR-fragments of *35S-GFP-NOS* from plasmid *p35S*-*GFP*. To remove residues of template plasmid DNA from the PCR-fragments, we purified PCR-fragments *35S-GFP-NOS* through agarose gel extraction. Then, purified PCR-fragments *35S-GFP-NOS* were transformed into epidermal cells of *Onion* peels with the biolistic bombardment delivery system. To compare transformation efficiency, plasmid DNA *p35S-GFP* was transformed in parallel. For a negative control, we transformed PCR-fragments generated from the same template plasmid but not containing GFP sequences. GFP signal was compared in transformations with PCR-fragments and with plasmid DNA. Results showed that GFP fluorescent signal was detected in the transformation with *35S-GFP-NOS* or *p35S-GFP*, no GFP signal was detectable in the negative control (Figure S1). These results indicated that PCR-fragment based transient expression system (PCR-TES) could be used as an alternative way to assess gene expression in plant cells. Next, we compared the efficiency between PCR-fragments transformation and plasmids transformation. PCR-fragment *35S-GFP-NOS* and plasmid *p35S-Cherry* were cotransformed into epidermal cells of *Onion* peels. Results showed that cells expressing Cherry fluorescent signal (red) were also displaying GFP fluorescent signal (green) (Figure 1B), suggesting that transformation with PCR-fragments, instead of plasmid DNA, could be a substantial approach for transiently assessing a gene expression in plant cells.

In attempted to examine the applicability of PCR-TES for analyzing subcellular localizations of genes, three cellular markers were tested in PCR-TES. PCR-fragments of *YFP-mTn* (for showing actin filaments) [19], *NAG*-*YFP* (for labeling golgi apparatus) [20] and *ER-YFP* (for showing endoplasmic reticulum) [21] were cotransformed with their correspondent plasmids (*p35S-CFP-mTn, p35S-NAG-CFP and p35S-ER-CFP*) into epidermal cells of *Onion* peels and *Arabidopsis* leaves, respectively. Results demonstrated that PCR-fragments (showing YFP fluorescence) of each cellular marker were expressed as efficient as their correspondent plasmids (showing CFP fluorescence) at their cellular locations (Figure 1C).

### Transient Expression of PCR-fragments in Protoplasts

**Figure 2:**
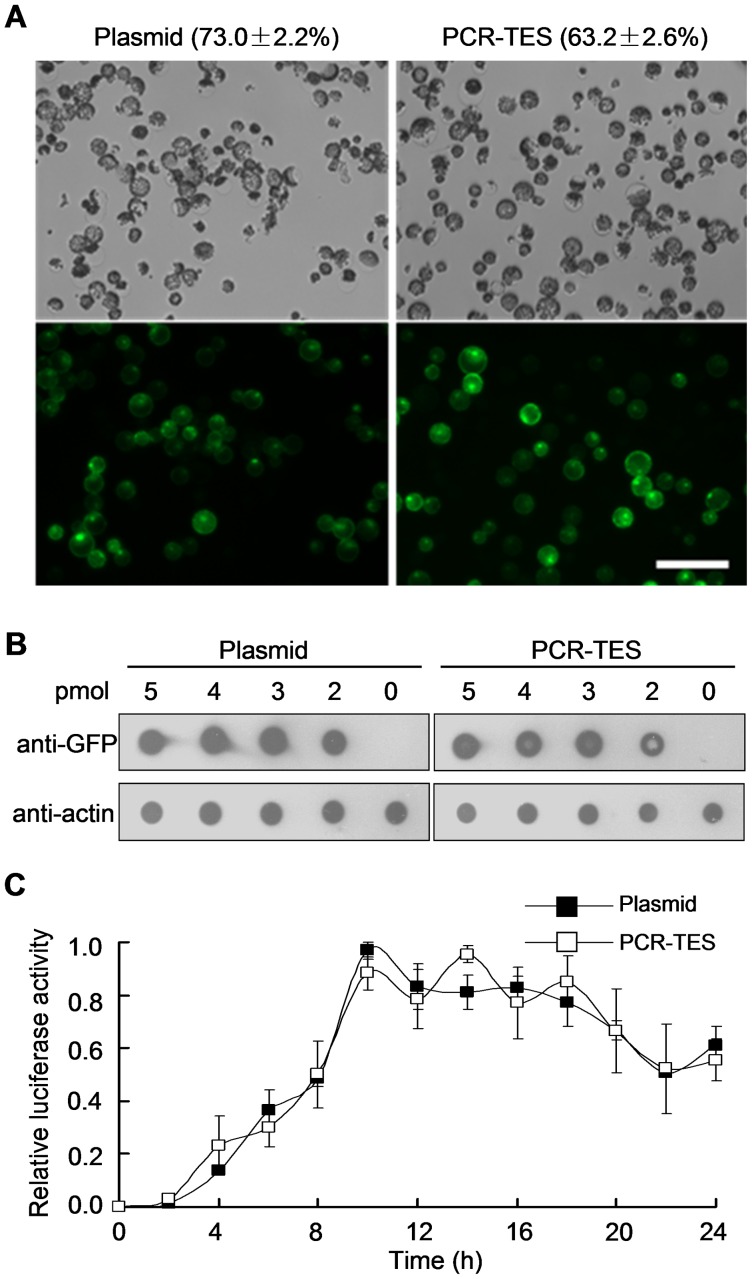
Transient expression of PCR-fragments in *Arabidopsis* mesophyll protoplasts. (A) Protoplasts were transfected with plasmid *p35S-GFP* (Plasmid) or with PCR-fragments *35S-GFP-NOS* (PCR-TES). Transformation efficiencies were determined by analyzing the protoplasts with fluorescence after incubation for overnight. Data represent the means±SEM from repeated experiment (n = 4). Scale bar, 200 µm. (B) Comparisons of transformation efficiencies at the protein level with the dot blot assay. A serial concentrations (5 pmol, 4 pmol, 3 pmol, 2 pmol and 0 pmol) of *p35S-GFP* (Plasmid) or PCR-fragments of *35S-GFP-NOS* (PCR-TES) were used to titrate out the transformation efficiency. Dots were blotted with anti-GFP antibody. The anti-actin shows the internal control. (C) Analysis on the stability of PCR-fragments in cells. Equal amount of *pUBQ10-LUC* (Plasmid) or PCR-fragments of *UBQ10-LUC* (PCR-TES) was transformed into 3×10^4^ protoplasts and aliquot into 12 samples. Protoplasts were harvested in 2 hours apart and the luciferase activity was measured. The relative luciferase activity was calculated as the ratio against to the maximum value. Data represent the means±SEM from repeated experiment (n = 3).

The transient expression approach to analyze a gene expression in mesophyll protoplasts of *Arabidopsis* has been efficiently applied for the cell-based assay [6]. To test the practicality of PCR-TES in protoplasts, we compared transformations with plasmid DNA *p35S-GFP* and with PCR-fragment *35S-GFP-NOS* in Col mesophyll protoplasts, respectively. Similar transformation efficiency was obtained with both transformation systems (Figure 2A, Figure S2A). Reproducible results were scored in repeated experiments. For instance, 63.2±2.6% transformation efficiency was obtained in PCR-TES whereas 73.0±2.2% transformation efficiency was shown in transformation with plasmid DNA (Table S1). To further confirm the transformation efficiency at protein level the dot blot analysis was performed (Figure 2B). We titrated out the amount of transformed PCR-fragments *35S-GFP-NOS* and plasmid DNA in a serial concentration (0 pmol, 2 pmol, 3 pmol, 4 pmol, and 5 pmol). After transformation and incubation for 8 hours, cell lysates were extracted and detected with anti-GFP antibody. Results showed the fact that expression level of GFP protein in PCR-TES and in plasmid transformation system was comparable (Figure 2B). In addition, we tested PCR-fragments directly generated using the fusion PCR method [22]. As shown in Figure S2B, the transformation of PCR-fragments *35S-GFP-NOS* generated through fusion PCR was comparable to that of using PCR-fragments *35S-GFP-NOS* amplified from template plasmid *p35S-GFP* (Figure S2B). To attest PCR-fragments that directly generated from genomic DNA without plasmid construction, we amplified the PCR-fragment *SnRK2.6* (*G-SnRK2.6*), containing its native promoter sequence, from genomic DNA of 2-week-old *Arabidopsis* seedlings (Figure S2C). The PCR-fragments *SnRK2.6* (*G-SnRK2.6*) were transformed into mesophyll protoplasts of Col, and results showed that increased expression level of *SnRK2.6* was detected in the protoplasts transformed with PCR-fragments *SnRK2.6* (*G-SnRK2.6*). In addition, the improved expression level of *RD29B* (an ABA responsive gene) was scored with and without ABA (5 µM) treatment; the enhanced relative activity (LUC/GUS) of *RD29B-LUC* was also measured (Figure S2C). Taken together, these data demonstrate that PCR-TES is applicable for examining a gene expression cassette (with its native promoter) without plasmid construction. To further explore how effective of PCR-TES at the protein level, we validated PCR-TES by transforming PCR-fragments of myc-tagging PYR1. PCR-fragments *35S-myc-PYR1-NOS* were expressed abundantly in protoplasts, as efficient as that shown in the transformation with plasmid *p35S-myc-PYR1* (Figure S2D). Collectively, these results provide the evidences in that PCR-TES could be practical for speedily examining gene expression in protoplasts.

To evaluate how stable PCR-fragments might be sustained in plant cells after transformation, we quantified activity of luciferase reporter (LUC) timely. Equal amount of PCR-fragments *UBQ-LUC-NOS* and plasmid DNA *pUBQ-LUC* was respectively transformed into mesophyll protoplasts. Results indicated that comparable expression patterns were produced in both transformation systems (Figure 2C). The LUC activity was detectable after transformation for 24 hours, indicating that PCR-fragments were as stable as plasmid DNA after being transformed into protoplasts.

### Evaluations of PCR-TES

**Figure 3:**
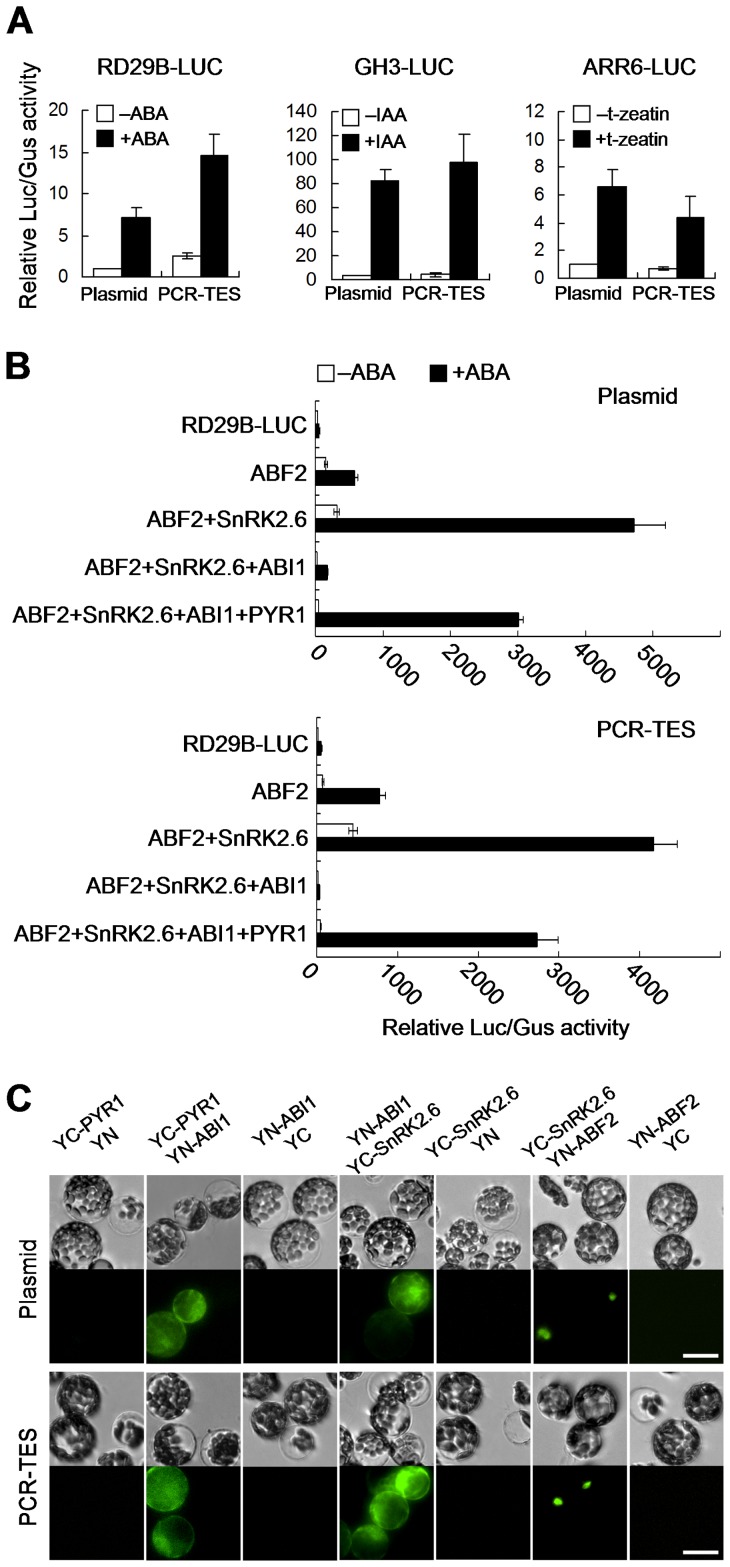
Comparisons of transformations with plasmid DNA and PCR fragments. (A) PCR-fragments (*RD29B-LUC-NOS, GH3-LUC-NOS* and *ARR6-LUC-NOS*) were transformed into protoplasts, respectively. Their correspondent plasmids were used for positive control experiments. Plasmid *pUBQ10-GUS* was cotransfected as the internal control (ABA, 5 µM; IAA, 1 µM; t-zeatin, 10 nM). Data represent the means±SEM from repeated experiments (n = 3). (B) The reconstitution of ABA signaling pathway with PCR-TES. Mesophyll protoplasts of Col were transformed with plasmids (Plasmid), or with PCR-fragments (PCR-TES). Plasmids *pRD29B-LUC* and *pUBQ10-GUS* were used as the ABA-responsive reporter and the internal control, respectively. After transformation, protoplasts were incubated for 5 hours in the absence of ABA (−ABA) or in the presence of 5 µM ABA (+ABA). Data represent the means±SEM from repeated experiments (n = 4). (C) Interactions of PYR1-ABI1, ABI1-SnRK2.6, and SnRK2.6-ABF2 were analyzed with BiFC assay. Plasmids (*p35S-YC-PYR1*, *p35S-ABI1-YN*, *p35S-YC-SnRK2.6* and *p35S-YN-ABF2*) and correspondent PCR-fragments were transformed into protoplasts. YC: C-terminal of YFP; YN: N-terminal of YFP. Scale bar, 30 µm.

To compare the applicability of PCR-TES versus the plasmid transformation system, we analyzed expression levels of several phytohormone responsive genes, such as *RD29B* (ABA response), *GH3* (auxin response), and *ARR6* (cytokinin response). Promoter sequences of these selected genes were fused to the reporter luciferase (LUC). PCR-fragments *RD29B-LUC-NOS*, *GH3-LUC-NOS* and *ARR6-LUC-NOS* were respectively transformed into protoplasts. In parallel, correspondent plasmid DNA of *pRD29B-LUC*, *pGH3-LUC*, or *pARR6-LUC* was also transformed. After treated with individual stimulus for 5 hours, the relative LUC/GUS activity was quantified. Despite of transforming with PCR-fragments or with plasmid DNA, similar expression patterns were scored (Figure 3A). Hence, these data support our suggestion on that PCR-TES might be used for assessing the expression level of a gene in plant cells. Thus, we designed experiments to reassemble the ABA signal transduction pathway that has been reported previously [11]. Equal amount of plasmids and PCR-fragments were transformed into Col mesophyll protoplasts, respectively. The relative LUC/GUS activity was quantified and consistent results were shown in both transformation systems (Figure 3B). In the presence of ABA, SnRK2.6 activated ABF2 was inhibited when ABI1 was added to the system; however, the inhibitory effect of ABI1 could be relieved by PYR1 (Figure 3B). To confirm this data, we examined the protein-protein interactions using PCR-TES and plasmid transformation system, respectively. PCR-fragments of *YC-PYR1* and *YN-ABI1*, *YN-ABI1* and *YC-SnRK2.6*, *YC-SnRK2.6* and *YN-ABF2* were transformed into protoplasts, respectively. The recombinant YFP signal was analyzed in each group of transformation (Figure 3C). In parallel, PCR-fragments of *35S-YC-NOS* (YC) and *35S-YN-NOS* (YN) were transformed into protoplasts and used for the negative control experiment (Figure 3C). Similar results were obtained in transformations with correspondent plasmids (Figure 3C). Together, our data are supportive to the fact that PCR-TES could facilitate the assessment of signaling molecules.

### Screen of CDPK Candidates Involving in the ABA Signaling

**Figure 4:**
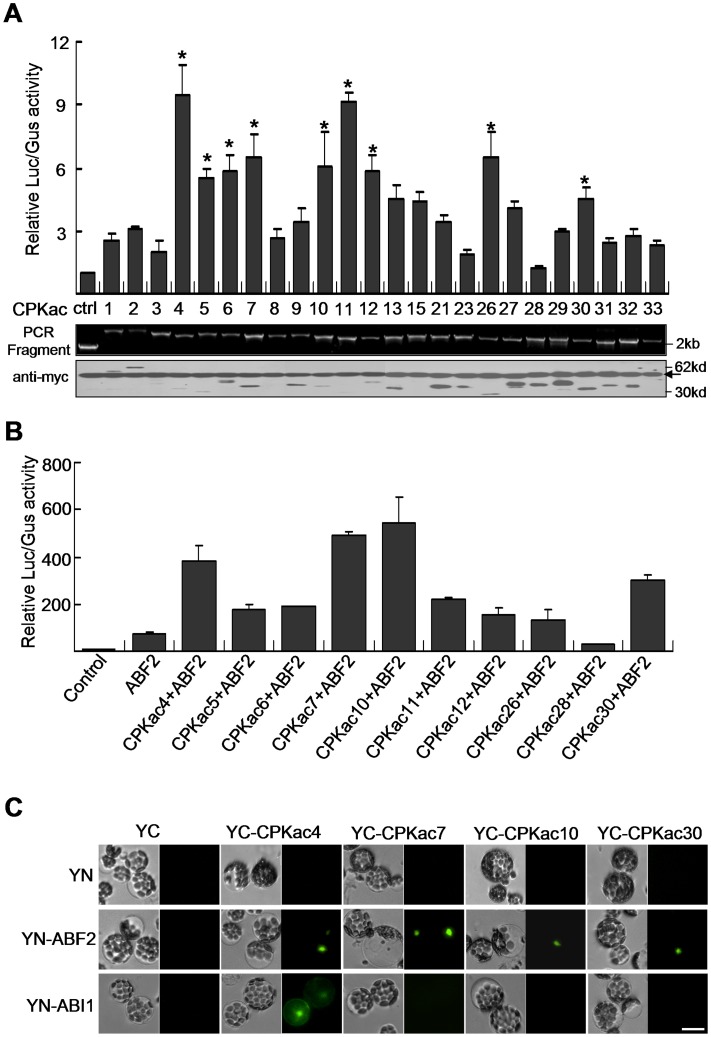
Functional analyses on CDPKs involving in the ABA signaling pathway. (**A**) Screening for the CPKac that may activate ABA-responsive *RD29B-LUC*. Protoplasts of Col were transformed with PCR-fragments of individual CPKac. Transformation of *35S-YFP-NOS* was used as the control (ctrl). Plasmids *pRD29B-LUC* and *pUBQ10-GUS* were used as the ABA-responsive reporter and the internal control, respectively. Relative LUC/GUS activity was measured after 5-hour incubation. The anti-myc antibody was used to show the expression level of CPKac. Arrow indicates non-specific protein band detected by anti-myc antibody. Asterisks (*) indicate 9 CPKac candidates possessed over five-fold expression levels of relative LUC/GUS activity. Data represent the means±SEM from repeated experiments (n = 4). (B) Analysis on CPKac and ABF2 in triggering *RD29B-LUC* activity in protoplasts. PCR-fragments of *ABF2* and *CPKac* were cotransfected to mesophyll protoplasts isolated from Col leaves. Plasmids *pRD29B-LUC* and *pUBQ10-GUS* were used as the ABA-responsive reporter and internal control, respectively. Data represent the means±SEM from repeated experiments (n = 3). (C) BiFC assay to examine the interactions of CPKac and ABF2 or ABI1 in PCR-TES. PCR-fragments (YN-ABF2, YN-ABI1 and YC-CPKac) were transformed into protoplasts. YC: C-terminal of YFP; YN: N-terminal of YFP Scale bar, 30 µm.

In order to examine how practical of PCR-TES could be, we designed a functional screen looking for CDPK (calcium-dependent protein kinase) family members which might play the role in the ABA signaling. We tested 24 members of CDPK which were reported to show predominant expression patterns in *Arabidopsis* leaves [12]. The constitutive activation form of each CDPK (CPKac) was generated by deleting the C-terminal Ca^2+^ regulatory domain and auto-inhibitory domain [12]. PCR-fragments composing c-myc tagged CPKac (Figure S3A) were transformed into protoplasts along with plasmid *pRD29B-LUC* (ABA-responsive reporter construct). In the absence of ABA, 9 out of 24 *CPKac*, such as *CPKac4*, *CPKac5*, *CPKac6*, *CPKac7*, *CPKac10*, *CPKac11*, *CPKac12*, *CPKac26* and *CPKac30*, could trigger activity of *RD29B-LUC*; more than five-fold increase in relative activity of *RD29B-LUC* was scored (Figure 4A).

It has been reported that some family members of CDPKs can phosphorylate ABF1 and ABF4 [23]. We attempted to test the role of screened 9 CPKac candidates in the regulation of ABF2, a bZIP transcription factor that has been reported to be one of players in response to ABA [24]. Results showed that synergistic effects in activating *RD29B-LUC* were produced when *ABF2* was coexpressed. Significant increase in relative activity of *RD29B-LUC* was observed in coexpressions with *CPKac4*, *CPKac7*, *CPKac10* and *CPKac30* (Figure 4B). To explore the correlation between CPKac and ABF2, we examined interactions between ABF2 and CPKac4, CPKac7, CPKac10, or CPKac30, respectively. The control experiment was to analyze the interaction between SnRK2.6 and ABF2 [11]. As results, each examined CPKac exhibited ability to interact with ABF2 in the nucleus. (Figure 4C and Figure S3B). Thus, our data implicated the notion that the regulation of CPKac to ABF2 (or other bZIP transcriptional factors) might be executed in the nucleus. Additionally, we attested the interaction of ABI1 and CPKac, and results suggest that CPKac4 is possible to interact with ABI1 (Figure 4C). To verify the reliability of results from PCR-TES we repeated all analyses in transformations with plasmids DNA. Not surprising, consistent results were reproduced (Figure S3C). Overall, our results not only prove the credibility of PCR-TES, but also suggest that CPKac4 might be a target or serves as an interactive molecule to ABI1.

### Reconstitution of CPK4-mediated ABA Signaling Pathway with PCR-TES

**Figure 5:**
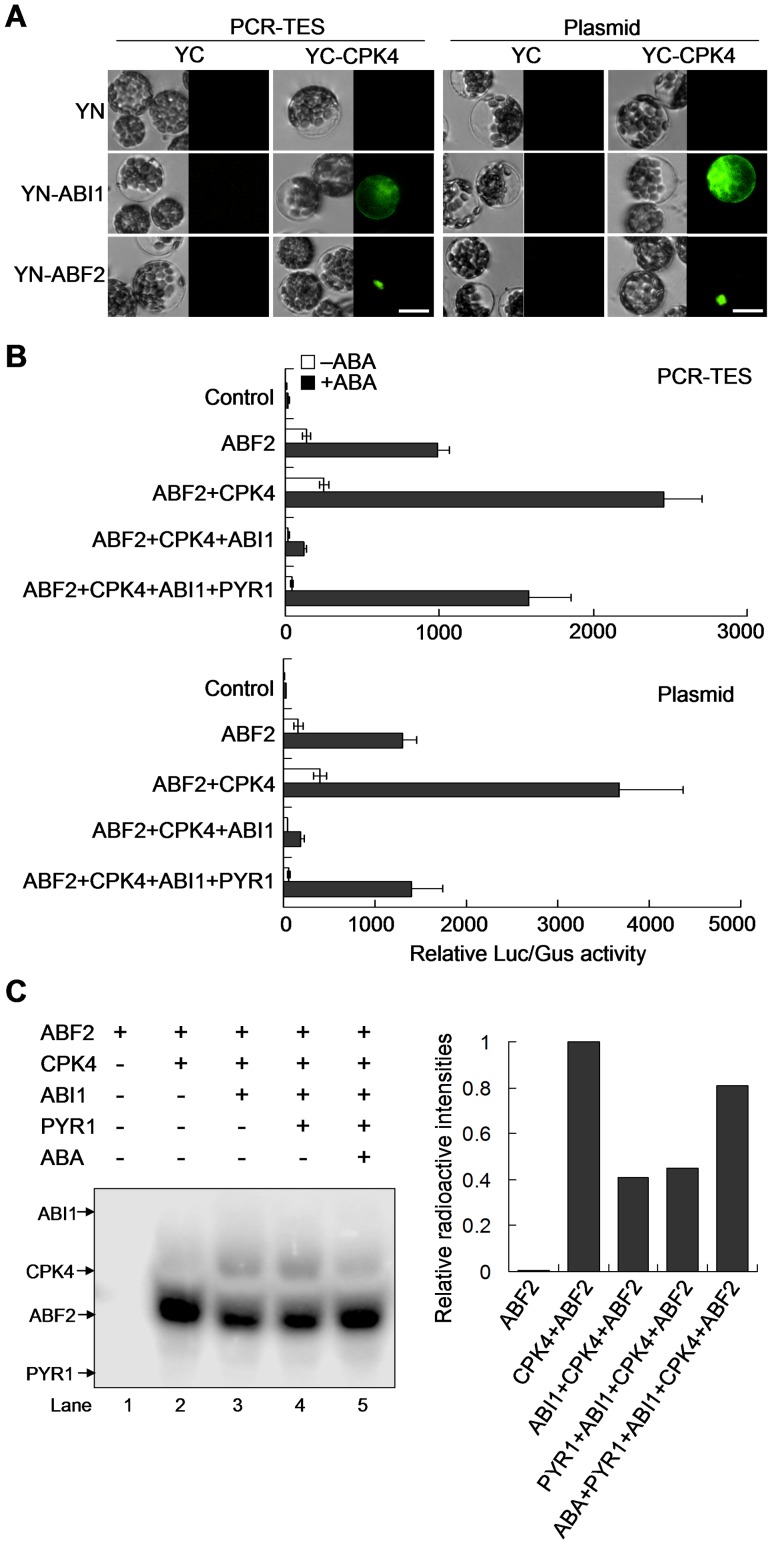
Analysis on *CPK4* function in the ABA signaling pathway. (A) Analyzing the interactions of CPK4 and ABI1 or ABF2 with PCR-TES and with the plasmid transformation system in protoplasts. YC: C-terminal of YFP; YN: N-terminal of YFP. Scale bar, 30 µm. (B) *CPK4* involving in ABA response was characterized in both systems (PCR-TES, Plasmid). Plasmids *pRD29B-LUC* and *pUBQ10-GUS* were used as the ABA-responsive reporter and the internal control, respectively. After transformation, protoplasts were incubated for 5 hours in the absence of ABA (−ABA) or in the presence of 5 µM ABA (+ABA). Data represent the means±SEM from repeated experiments (n = 4). (C) Phosphorylation of CPK4 to ABF2 was demonstrated with *in vitro* kinase assay (left panel). Relative radioactive intensities of γ-^32^P were quantified (right panel) with Typhoon 9200 Imager.

To determine the role of *CPK4* in the ABA signal transduction pathway, we focused on examining the full length *CPK4*. Consistent result was obtained in PCR-TES and plasmid transformation system. The interaction between CPK4 and ABF2 was observed in protoplasts (Figure 5A). In ABA treatment, *CPK4* and *ABF2* could synergistically affect *RD29B-LUC* activity. The relative activity (LUC/GUS) of *RD29B-LUC* was enhanced more than 2000-fold in the presence of ABA. In contrast, the relative activity (LUC/GUS) of *RD29B-LUC* was strikingly inhibited by *ABI1*; however, the inhibitory effect of *ABI1* could be rescued by *PYR1* upon ABA treatment (Figure 5B). These results were further confirmed in transformation with correspondent plasmids (Figure 5B). To investigate the role of *CPK4* in modulating *ABF2* activity while responding to ABA, recombinant proteins of His-PYR1, His-ABF2, His-CPK4 and GST-ABI1 were prepared (Figure S4) and analyzed (Figure 5C). Undertaken the *in vitro* kinase assay, we found out that CPK4 could phosphorylate ABF2. The ABI1 inhibitory effect on CPK4 kinase activity could be partially compromised by PYR1 and eventually reversed by ABA (Figure 5C). In addition, we noticed that self-phosphorylation of CPK4 was phenomenal when ABI1 was present in the system (Figure 5C, lane 3), which was similar to that observed in analyzing CPK23 [13]. Taken together, our results provide supportive evidences on that PCR-TES is applicable for rapidly screening molecular candidates and for analyzing molecular mechanisms underlying the ABA signal transduction.

## Discussion

### PCR-TES can be a Pivotal Tool for the Cell-based Assay

For studying regulations of genes in plant cells, diverse approaches are undertaken at desired circumstances. In this report, we introduce a simple system that can be used for quickly examining a gene function in plant cells. We demonstrated that PCR-TES (PCR-fragment based transient expression system) could facilitate preliminary screening for putative candidates in the transient expression assay. The transformation efficiency of PCR-TES can be comparable to that of conventional plasmid transformation.

Aiming at quick screen and fast evaluation of putative candidates, PCR-TES possesses its advantage. Directly transforming PCR-fragments which are amplified from an existing template plasmid or from a source of DNA library through fusion PCR or from a source of genomic DNA into plant cells could shorten experimental procedures, because we do not have to construct or purify plasmids before conducting a preliminary screen. In conventional plasmid transformation system, over 50% transformation efficiency is required for obtaining a reliable analytic result [6]. Traditional CsCl-gradient purification is used to be the way to purify large amount of high quality plasmid DNA; however it can be time consuming and troublesome [6]. Using routine plasmid preparation kit, one may be able to prepare large amount of plasmid DNA, but it might result in low transformation efficiency and unsatisfied for obtaining steady results [25] (Figure S2A). One of the plasmid midi-preparation kits (Plasmid Plus, QIAGEN, Germany) has been suggested for purifying high quality plasmids [25]. However, comparing to directly transforming PCR-fragments it would multiply experimental procedures, especially when we need to handle dozens of samples for preliminary screening. Thus, PCR-TES may serve as an alternative way to speedy up screen of putative molecules before doing extensive characterizations. In addition, in some cases when the cloned gene is harmful to *E. coli* growth, plasmid construction or propagation in *E. coli* can be problematic [26], [27]. Under these circumstances, the PCR-TES can be a way used for bypassing this problem.

Direct delivery of PCR-fragments into cells is time saving. After brief purification to remove solvents of PCR reaction, PCR-fragments are ready for transformation. PCR-TES might be feasible for conducting high throughput screening; at least, it is usable for analyzing molecular candidates. In our analyses, PCR-fragments were amplified from the template plasmid DNA. Contamination of template plasmid DNA was a concern. We clarified this fear by comparing transformations with PCR-fragments prepared from non-coding sequences of the same template plasmid. Results were cleared out this concern (Figure S1). Another argument would be the stability of transformed PCR-fragments in cells. To address this point we examined transformation efficiency at the protein level and found out that PCR-fragments were as effective as plasmid DNA in plant cells after transformation (Figure 2 and Table S1). In general, the accuracy of plasmid constructs has to be confirmed by DNA sequencing. Only the confirmed DNA sequences can be constructed into a vector for making the correct plasmid. If the mutated DNA sequences were inserted into a vector and then transformed into a carrier such as *E. coli*, the resulted bacterial colonies would be harboring the mutations; as a consequence, all the propagated plasmids from that single colony of *E. coli* would also carry mutations which may cause a general effect. In PCR-TES, however, PCR-fragments are amplified from a correct plasmid template, a source of DNA library through fusion PCR [22] or a source of genomic DNA with its native promoter; among the millions of PCR fragments in a PCR reaction, mutated PCR fragments may be minor. Nobody can “assume” that such mutations will never cause a general effect. Therefore, we always compare results from the PCR-TES to those from the conventional plasmid transformations (Figure 3, Figure 5 and Figure S2). Our data indicate that consistent results were always produced in both systems.

Although PCR-TES may facilitate massive screening for candidates, its application for stable transformation is unsatisfied. If a large-scale transformation is needed, PCR-TES might not be satisfied for conducting such experiment in considering the cost of DNA polymerases for amplification of PCR-fragments. A compromise way one might chose is to scale up the PCR reaction. In fact, we found out that 100–200 µg PCR-fragments could be easily generated by scale up the PCR reaction at a relatively lower cost. In our point of view, combining PCR-TES and plasmid transformation system may accelerate our pace in studying gene regulation in plant cells.

### Application of PCR-TES in Speeding Up Screen of Putative ABA Responsive CDPKs

Thirty-four family members of calcium-dependent protein kinases (CDPKs) are found in *Arabidopsis* genome. Some CDPK members involving in ABA- and/or abiotic-signal transduction pathways have been determined. For example, *CPK10*, *CPK23* and *CPK32* are involved in ABA- or drought-responses [28]–[30]. In addition, *CPK3* and *CPK6* are key regulators in the ABA-regulated stomatal movement. ABA induced stomatal closure and activation of slow-type anion channels are inhibited in *cpk3* and *cpk6* mutants [31]. Functional as kinases, CPK4, CPK11 and CPK32 can regulate activity of ABF1 or ABF4, the bZIP transcription factor that responds to ABA through phosphorylation modification [23], [28]. Moreover, CPK21 and CPK23 can modulate activity of the anion channel protein SLAC1 in ABA-induced stomatal movement [13]. In this report we revealed the potential role of CPK7 and CPK26 in ABA response which was not reported in previous studies [23], [28]–[32]. In our screen, we could determine that CPK7 and CPK26 were able to trigger *RD29B-LUC* activity using PCR-TES (Figure 4A), demonstrating that PCR-TES can be a practical tool for quickly testing putative signaling molecules. In PCR-TES, we were able to determine the functional specificity of individual CDPK in regulation of ABF2 activity. Although CPK4 and CPK11 both share high identity in amino acid sequences, only CPK4 (not CPK11) could effectively influence ABF2 activity (Figure 4B). In contrast to other CDPKs, CPK28 was likely to play an opposite role in regulating ABF2 activity (Figure 4B); suggesting that the subgroup IV CDPK members (CPK16, CPK18, and CPK28) [33] may play differential role in comparison to other CDPK members. The interaction of CPK23 and ABI1 is shown with BiFC assay in *Xenopus oocytes* [13]. Here we could demonstrate that interaction between CPK4 and ABI1 was observed in protoplasts in PCR-TES (Figure 4C and Figure 5A). In addition, CPK4 could synergistically induce the *RD29B-LUC* activity in the presence of ABF2 (Figure 5B). These results support the importance of CDPKs in the ABA signal transduction pathway.

Upon ABA stimulation, the receptor PYR/RCAR recruits ABI1 (PP2C) [34], [35], thus the suppression of ABI1 on kinase SnRK2.6 can be relieved; in turn, the ABA signaling is relayed *via* modulating ABF2 activity [11]. Based on this theory, we were able to reconstitute the ABA response in protoplasts with PCR-TES. We attested the correlation between phosphorylation of CPK4 and activation of ABF2 *in vivo* and *in vitro*. Moreover, we unveiled the negative regulation of ABI1 to CPK4 kinase activity, which is similar to the negative regulatory fashion of ABI1 to SnRK2.6 [11]. Again, the data from reconstitution of the ABA signaling in protoplasts and the *in vitro* kinase assay are evidently confirmed the practicality of PCR-TES.

## Materials and Methods

### PCR-fragments Preparation

PCR-fragments were generated in three different ways. First, PCR-fragments were directly generated from correspondent plasmids with the universal primer pair PVU-F (5′-CTGGCACGACAGGTTTCCCGACT-3′) and PVU-R (5′-GGCGAAAGGGGGATGTGCTGCAA-3′) through PCR amplification in 35-cycles, and the polymerase KOD-Plus-Neo (Toyobo, Japan) was used. Produced PCR-fragments were purified by following the protocol of phenol/chloroform extraction and ethanol precipitation [18]. Briefly, equal volume of phenol/chloroform (1∶1) was added to the PCR mixture. The aqueous phase was collected after centrifugation (12,000 g) for 3 minutes and then mixed with 1/10 volume of sodium acetate (3 M, pH 5.2) and 2.5 volume of ice-cold 100% ethanol. After centrifugation (12,000 g) for 5 minutes, the DNA of PCR-fragments was precipitated. Through a brief washing with 70% ethanol, the DNA pellet of PCR-fragments was dissolved in water and quantified. In general, about 10–15 µg PCR-fragments could be yielded from a 50 µl PCR reaction and the final concentration of purified PCR-fragments was adjusted to 1 µg/µl in water or TE buffer and stored at −20°C. To remove residues of template plasmid DNA from the PCR-fragments, we purified PCR-fragments through agarose gel extraction (TIANgel Midi Purification Kit, Tiangen, China). Next, PCR-fragments were produced using fusion PCR by following the method ascribed in the previous report [22] with minor modifications. In brief, fragments *35S* and *NOS* were amplified from plasmid *p35S-MCS* (kindly provided by Dr. Benedikt Kost, University of Erlangen-Nuremberg, Germany) using primer pairs Fu35S-F/Fu35S-R and FuNOS-F/FuNOS-R, respectively. CDS sequence of GFP was amplified from plasmid *p35S-GFP*, that was generated by inserting GFP fragment into *p35S-MCS*, using primer pair FuGFP-F/FuGFP-R. After agarose gel extraction, PCR-fragments *35S-GFP-NOS* were assembled using the two-step fusion PCR method [22]. Purified PCR-fragments could be stored up to six months in TE buffer at −20°C. The third way to make PCR-fragments is directly amplifying a gene fragment from the genomic DNA that was extracted from 2-week-old seedlings of *Arabidopsis*. For instance, PCR-fragments of *SnRK2.6* (*G-SnRK2.6*, with its native promoter) were thus generated. All primer sequences for producing PCR-fragments are listed in Table S2.

### Biolistic Bombardment Assay

PCR-fragments or plasmids were delivered through the biolistic bombardment method (Bio-Rad, USA). Young leaves from 3-week-old *Arabidopsis* plants or epidermal peels from *Onion* were used in this assay. In each experiment, 2 µg PCR-fragments and/or 2 µg plasmid DNA were mixed with 60 mg/ml gold particles (diameter in 1.0 µm, Bio-Rad) in 25 µl coating buffer that contains 1 M CaCl_2_, and 16 mM spermidine. The detailed protocol for the biolistic bombardment was described in previous reports [8], [9]. After bombardment transformed leaves were incubated for overnight under light conditions at 23°C and observed with NIKON TE-2000U fluorescent microscope (Nikon, Japan) and Olympus Confocal Laser Scanning Microscope FV1000 (Olympus, Japan).

### Transient Expression Assay in Mesophyll Protoplasts

Protoplasts were isolated from leaves of 4-week-old Col plants and transformed using the method ascribed in the previous report [6] with some modifications, in order to suite for transforming PCR-fragments. First, 1×10^4^ protoplasts in 50 µl volume were mixed with plasmids DNA (in total 10 µg) or PCR-fragments (in total 10 µg); then, gently mixed with 60 µl PEG solution (40% W/V PEG-4000, 0.2 M mannitol, 100 mM CaCl_2_). After 10 minutes incubation at room temperature, 240 µl W5 solution (2 mM MES at pH 5.7, 54 mM NaCl, 125 mM CaCl_2_, 5 mM KCl) was added to the mixture to stop the transformation. Protoplasts were resuspended in 250 µl WI solution (4 mM MES at pH 5.7, 0.5 M mannitol, 20 mM KCl) and incubated at 23°C.

For the luciferase assay, protoplasts were harvested after 5-hour incubation under light conditions at 23°C with or without stimuli (5 µM ABA, or 1 µM IAA, or 10 nM t-zeatin). The activities of LUC and GUS were measured with the GloMax-Multi Jr Single Tube Multimode Reader (Promega, USA) by following the protocol described previously [6]. In each sample, 1 µg plasmid pUBQ10-GUS was used as an internal control and 4 µg reporter plasmids or reporter PCR-fragments were used. All experiments were repeated at least three times. For BiFC assays, protoplasts were transformed with plasmids (5 µg) or PCR-fragments (5 µg) and incubated for overnight at 23°C. Images were acquired with NIKON TE-2000U fluorescent microscope (Nikon, Japan).

### Protein blot

For dot blot assay, the lysate of protoplasts was extracted from transformed protoplasts in 20 µl lysis buffer (25 mM Tris-phosphate at pH 7.8, 2 mM DTT, 2 mM 1,2-diaminocyclohexane-N,N,N’,N’-tetraacetic acid, 10% glycerol, 1% Triton X-100). Then, extracted total protein was dotted and blotted on Hybond ECL Nitrocellulose Membrane (GE Healthcare) by following the manufacturer’s protocols. GFP was detected with antibody anti-GFP (Proteintech Group) whereas anti-actin (Proteintech Group) was used as the internal control. For western blot, the protoplast lysate was directly mixed with 50 µl loading buffer and separated with 12% SDS-PAGE gel. Antibody of c-myc (Sigma, USA) was used to detect the protein expression level.

### *In vitro* Kinase Assay

Recombinant proteins of His-PYR1, GST-ABI1, His-CPK4 and His-ABF2 were respectively expressed in *E. coli* and purified. The *in vitro* kinase assay was carried out according to the method described previously [12], [13] with some modifications. First, His-PYR1 (2 µg) and GST-ABI1 (2 µg) were incubated together with or without 1 µM ABA at room temperature (25°C) for 5 minutes. Afterwards, His-CPK4 (1 µg) and His-ABF2 (5 µg) were added to the 20 µl reaction system (20 mM Tris-HCl at pH7.4, 100 mM NaCl, 12 mM MgCl_2_, 1 mM CaCl_2_, 1 µM ATP, 5 µCi of [γ-^32^P] ATP, and 1 mM DTT). After incubation for 30 minutes at 30°C, the reaction was stopped by adding 20 µl 2X loading buffer and separated with 12% SDS-PAGE gel. Radioactive intensities of γ-^32^P were measured with Typhoon 9200 Imager (GE healthcare, USA).

### Plasmids Construction

Plasmid *p35S-MCS-Myc* was constructed by inserting blunt ended fragment of c-myc tag into vector *p35S-MCS* that contains a 35S promoter, multiple cloning sites (MCS) and a NOS terminator (kindly provided by Dr. Benedikt Kost, University of Erlangen-Nuremberg, Germany). Plasmid *p35S-MCS-YFP* or *p35S-MCS-CFP* was also constructed by inserting CFP or YFP into vector *p35S-MCS*. Plasmids *p35S-YFP-mTn* and *p35S-CFP-mTn* were constructed by modifying plasmid *GFP-mTn* [19] through replacing GFP to CFP or YFP. Plasmid *p35S-NAG-YFP* was generated by replacing the CFP to YFP based on plasmid *p35S-NAG-CFP* (kindly provided by Dr. Jian Xu, Huazhong Agricultural University, China) [20]. Plasmid *p35S-ER-CFP* and *p35S-ER-YFP* was constructed by inserting the sequence of ER signal peptide [21] to *p35S-MCS-YFP* or *p35S-MCS-CFP*. Promoter *UBQ10* was amplified from plasmid *pUBQ10-GUS* [17] and then inserted into the *LUC* empty vector that was generated by deleting promoter *RD29A* fragment from plasmid *pRD29A-LUC*, therefore *pUBQ-LUC* was constructed. Plasmids *pUBQ10-GUS*, *pGH3-LUC* and *pARR6-LUC* were both obtained from ABRC (http://www.arabidopsis.org/) [17]. Plasmid *pRD29B-LUC* was constructed as described in the previous report [11]. The *CPKac* plasmids were constructed by inserting individual *CPKac* fragment, which was amplified from the cDNA library of Col seedlings with correspondent primers (Table S2), into the vector *p35S-MCS-Myc*. Plasmids *p35S-YC-MCS* and *p35S-YN-MCS* were constructed by following the method described previously [36]. Briefly, the C-terminal of EYFP (YC) or N-terminal of EYFP (YN) fragment was respectively inserted into the vector. For expressing recombinant protein in *E. coli,* CDS of *PYR1*, *CPK4* and *ABF2* were cloned into *pET28* vector (Novagen, USA) and *ABI1* was inserted into *pGEX-6P* vector (GE Healthcare, USA). Detailed information about primer sequences for plasmid construction can be found in Table S2.

## Supporting Information

Figure S1 Comparisons of transformations with plasmid DNA and with PCR-fragments in epidermal cells of *Onion*. Plasmid DNA of *p35S-GFP* or PCR-fragments *35S-GFP-NOS* was delivered into *Onion* epidermal cells using biolistic bombardment method. PCR-fragments (not containing GFP sequences) were amplified from the plasmid *p35S-GFP* and used as the negative control (Control). Images were acquired under microscope after an overnight incubation. Scale bar, 200 µm.
(TIF)

Figure S2 Comparisons of protein expression levels between transformations with plasmid DNA and with PCR-fragments. (A) Comparison of transformations efficiencies using PCR-fragments and plasmids which were prepared in different methods (Mini-1, Mini-2 and Mini-3 stand for mini-prepared plasmids; Maxi: maxi-prepared plasmids; CsCl: using CsCl prepared plasmids). Transformation efficiencies were quantified. Data represent the means±SEM from repeated experiment (n = 3). Scale bar, 200 µm. (B) Transient expression of PCR-fragment from fusion PCR. Left panel: Diagram (not in scale) to show construction of the PCR-fragment cassette using fusion PCR. Right panel: PCR-fragment *35S-GFP-NOS* was amplified from plasmid *p35S-GFP* (from plasmid) or using fusion PCR (from fusion PCR), and then transformed into protoplast. (Chloroplast: autofluorescence). Scale bar, 200 µm. (C) Transient expression of PCR-fragment from genomic DNA. PCR-fragment *SnRK2.6* was generated using genomic DNA of *Arabidopsis*, and then tested in transient expression assay. Upper panel: Diagram (not in scale) to show construction of *SnRK2.6* PCR-fragment cassette. PCR-fragment *SnRK2.6* (*G-SnRK2.6*) was transformed into Col protoplasts. After transformation, protoplasts were incubated for 6 hours without (−ABA) or with 5 µM ABA (+ABA). The gene expression level of *SnRK2.6* and *RD29B* was quantified using qRT-PCR. The ABA-induced *RD29B-LUC* activity was also quantified. Plasmid *pUBQ10-GUS* was used as the internal control. All data represent the means±SEM from repeated experiments (n = 3). (D) Plasmid *p35S-myc-PYR1* and PCR-fragments *35S-myc-PYR1-NOS* were transformed into protoplasts, respectively; a serial of dilution of protoplasts was titrated out for detecting myc-PYR1. Total proteins were extracted after 8-hour incubation. Protein expression level of myc-PYR1 was determined by western blot with c-myc antibody. Control, protein extract from protoplasts without transformation; Lane 1, 1∶20 dilution of prepared protoplasts (2X10^5^ protoplasts/ml); Lane 2, 1∶40 dilution of prepared protoplasts; Lane 3, 1∶80 dilution of prepared protoplasts.
(TIF)

Figure S3 Analysis on interactions in BiFC assay. (A) Diagram (not in scale) to show components of PCR-fragments of CPKac. (B) Subcellular localization for interactions between *CPKac or SnRK2.6 and ABF2* in BiFC assay. Plasmid *p35S-YC-CPKac4, p35S-YC-CPKac7, p35S-YC-CPKac10*, *p35S-YC-CPKac30 or p35S-YC-SnRK2.6* was cotransfected with *p35S-YN-ABF2* into protoplasts. *p35S-CFP-ABF2* was co-transformed to mark the nucleus [24]. Merge shows the colocalizations. YC: C-terminal of YFP; YN: N-terminal of YFP. Scale bar, 30 µm. (C) Interaction between *ABI1* or *ABF2* and *CPKac* assessed in BiFC assays. Plasmid *p35S-YC-CPKac4, p35S-YC-CPKac7, p35S-YC-CPKac10* or *p35S-YC-CPKac30* was transfected together with *p35S-YN-ABI1* or *p35S-YN-ABF2* into protoplasts, respectively. YC: C-terminal of YFP; YN: N-terminal of YFP. Scale bar, 30 µm.
(TIF)

Figure S4 Analyzing expressions of recombinant proteins. Recombinant proteins of His-PYR1, GST-ABI1, His-CPK4 and His-ABF2 were analyzed in 12% SDS-PAGE gel and shown in coomassie staining.
(TIF)

Table S1 Comparisons of transformation efficiencies in protoplasts.
(DOC)

Table S2 Primer sequences for plasmids constructions and qRT-PCR experiments.
(DOC)
