# Supplementary figures and images for: Directly Transforming PCR-Amplified DNA Fragments into Plant Cells Is a Versatile System That Facilitates the Transient Expression Assay
Source: PLoS One. 2013 Feb 26;8(2):e57171. doi: 10.1371/journal.pone.0057171 (PMC3582559; doi:10.1371/journal.pone.0057171)

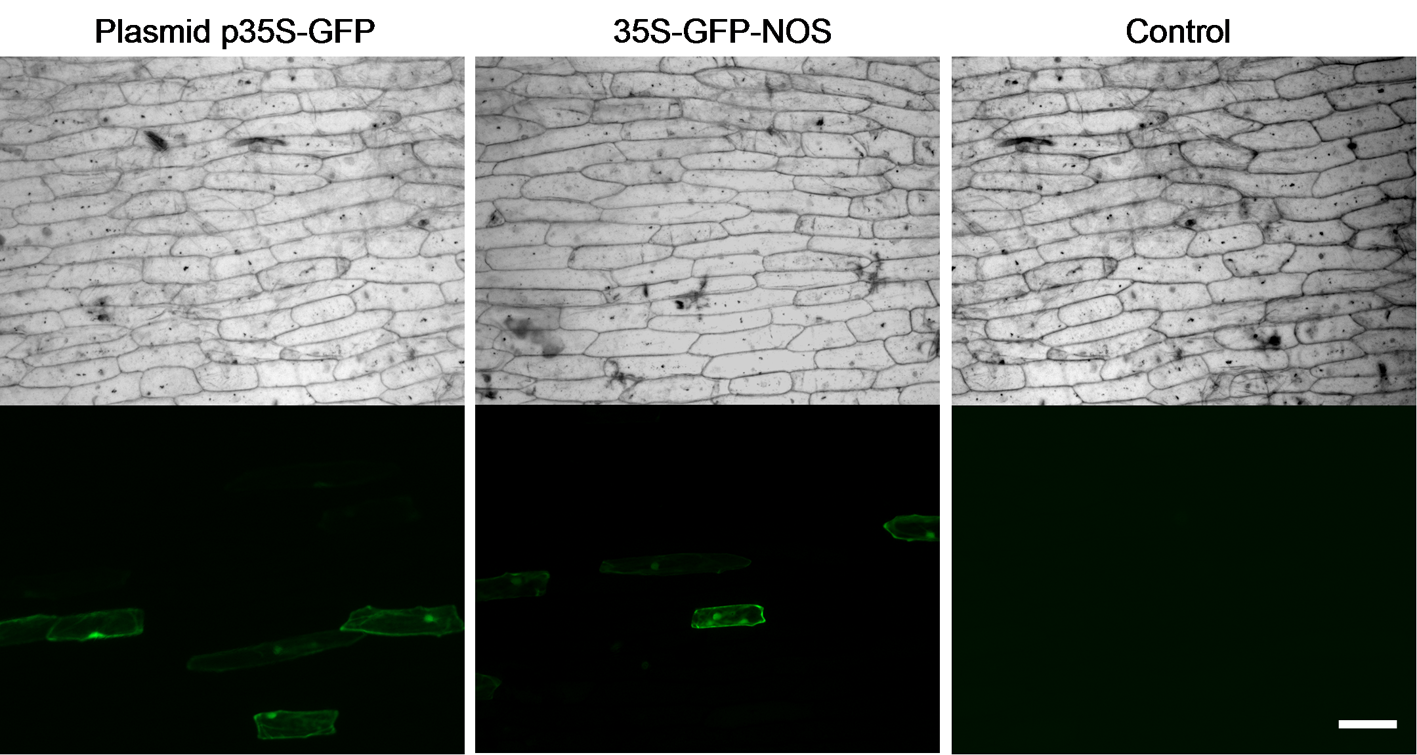

Supplement: Figure S1 — Comparisons of transformations with plasmid DNA and with PCR-fragments in epidermal cells of Onion. Plasmid DNA of p35S-GFP or PCR-fragments 35S-GFP-NOS was delivered into Onion epidermal cells using biolistic bombardment method. PCR-fragments (not containing GFP sequences) were amplified from the plasmid p35S-GFP and used as the negative control (Control). Images were acquired under microscope after an overnight incubation. Scale bar, 200 µm. (TIF) [file pone.0057171.s001.tif]

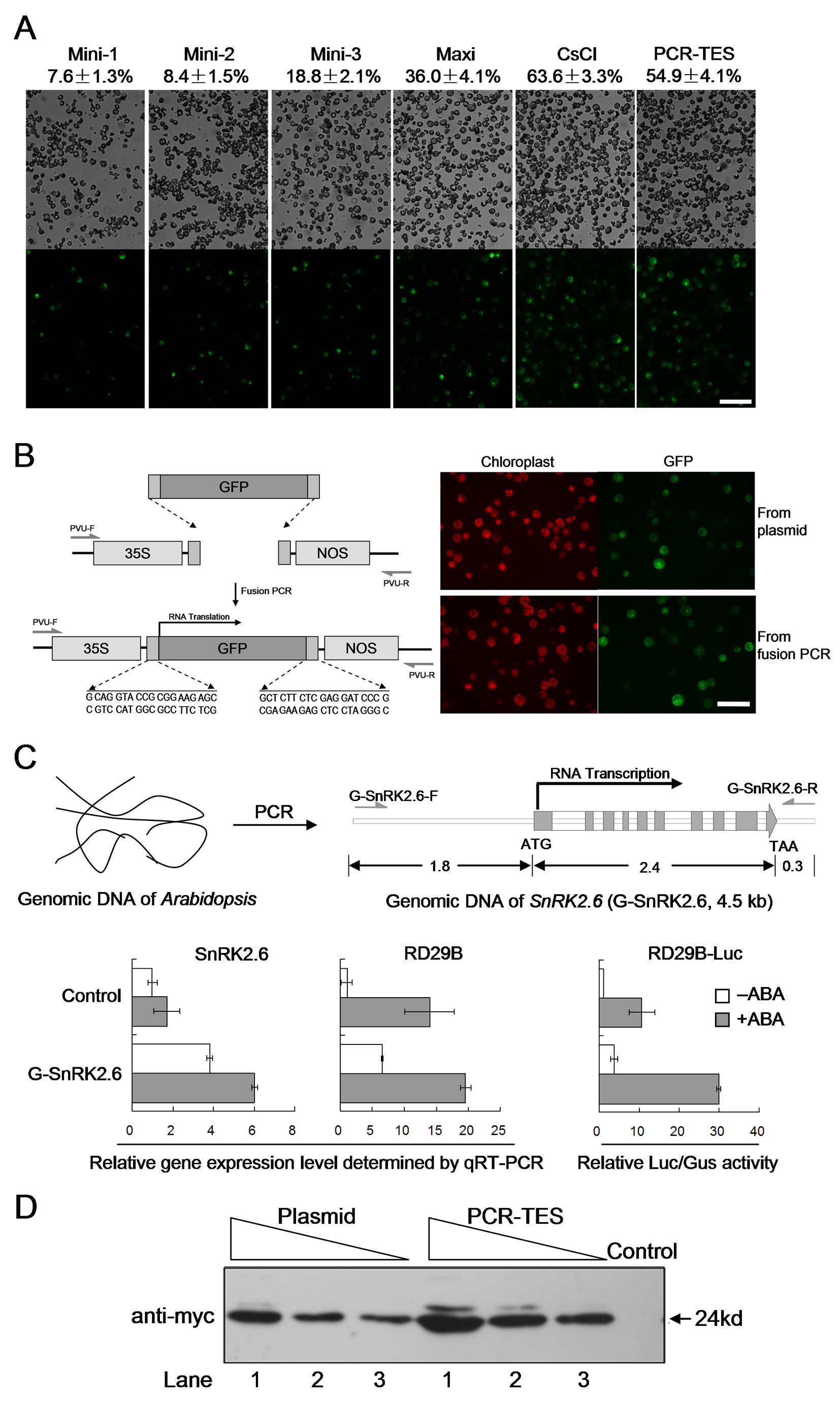

Supplement: Figure S2 — Comparisons of protein expression levels between transformations with plasmid DNA and with PCR-fragments. (A) Comparison of transformations efficiencies using PCR-fragments and plasmids which were prepared in different methods (Mini-1, Mini-2 and Mini-3 stand for mini-prepared plasmids; Maxi: maxi-prepared plasmids; CsCl: using CsCl prepared plasmids). Transformation efficiencies were quantified. Data represent the means±SEM from repeated experiment (n = 3). Scale bar, 200 µm. (B) Transient expression of PCR-fragment from fusion PCR. Left panel: Diagram (not in scale) to show construction of the PCR-fragment cassette using fusion PCR. Right panel: PCR-fragment 35S-GFP-NOS was amplified from plasmid p35S-GFP (from plasmid) or using fusion PCR (from fusion PCR), and then transformed into protoplast. (Chloroplast: autofluorescence). Scale bar, 200 µm. (C) Transient expression of PCR-fragment from genomic DNA. PCR-fragment SnRK2.6 was generated using genomic DNA of Arabidopsis, and then tested in transient expression assay. Upper panel: Diagram (not in scale) to show construction of SnRK2.6 PCR-fragment cassette. PCR-fragment SnRK2.6 (G-SnRK2.6) was transformed into Col protoplasts. After transformation, protoplasts were incubated for 6 hours without (−ABA) or with 5 µM ABA (+ABA). The gene expression level of SnRK2.6 and RD29B was quantified using qRT-PCR. The ABA-induced RD29B-LUC activity was also quantified. Plasmid pUBQ10-GUS was used as the internal control. All data represent the means±SEM from repeated experiments (n = 3). (D) Plasmid p35S-myc-PYR1 and PCR-fragments 35S-myc-PYR1-NOS were transformed into protoplasts, respectively; a serial of dilution of protoplasts was titrated out for detecting myc-PYR1. Total proteins were extracted after 8-hour incubation. Protein expression level of myc-PYR1 was determined by western blot with c-myc antibody. Control, protein extract from protoplasts without transformation; Lane 1, 1∶20 dilution of prepared protoplasts (2X1 [file pone.0057171.s002.tif]

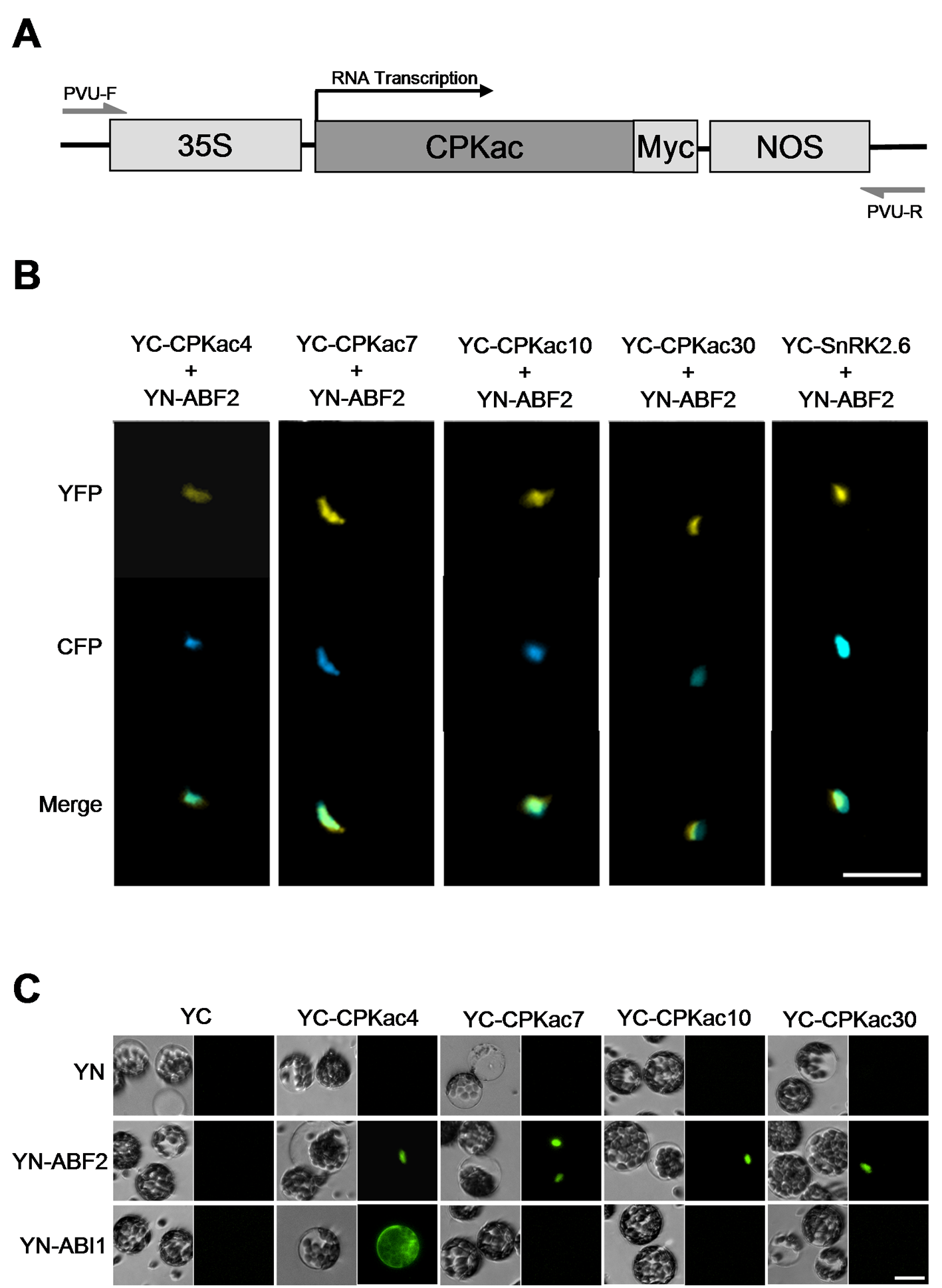

Supplement: Figure S3 — Analysis on interactions in BiFC assay. (A) Diagram (not in scale) to show components of PCR-fragments of CPKac. (B) Subcellular localization for interactions between CPKac or SnRK2.6 and ABF2 in BiFC assay. Plasmid p35S-YC-CPKac4, p35S-YC-CPKac7, p35S-YC-CPKac10, p35S-YC-CPKac30 or p35S-YC-SnRK2.6 was cotransfected with p35S-YN-ABF2 into protoplasts. p35S-CFP-ABF2 was co-transformed to mark the nucleus [24]. Merge shows the colocalizations. YC: C-terminal of YFP; YN: N-terminal of YFP. Scale bar, 30 µm. (C) Interaction between ABI1 or ABF2 and CPKac assessed in BiFC assays. Plasmid p35S-YC-CPKac4, p35S-YC-CPKac7, p35S-YC-CPKac10 or p35S-YC-CPKac30 was transfected together with p35S-YN-ABI1 or p35S-YN-ABF2 into protoplasts, respectively. YC: C-terminal of YFP; YN: N-terminal of YFP. Scale bar, 30 µm. (TIF) [file pone.0057171.s003.tif]

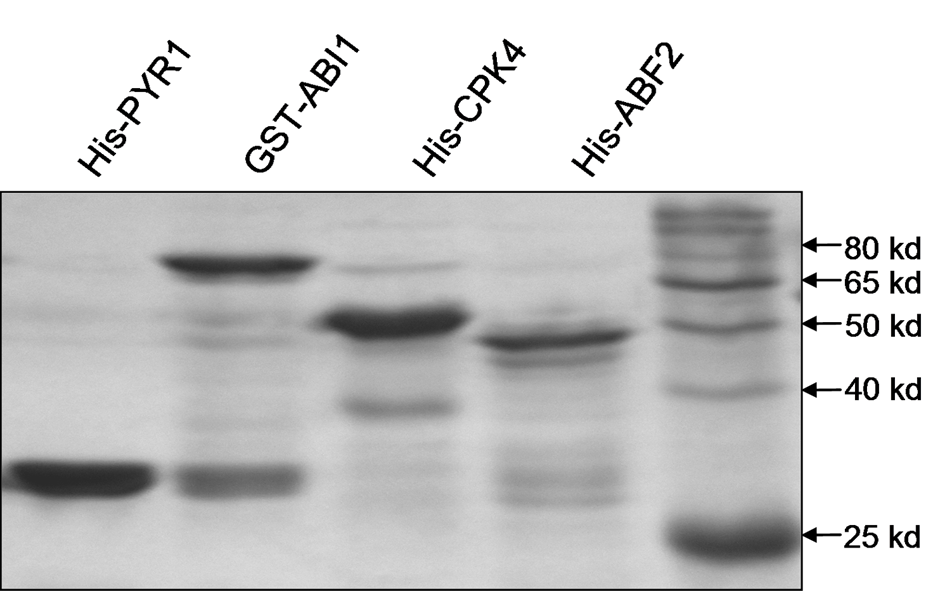

Supplement: Figure S4 — Analyzing expressions of recombinant proteins. Recombinant proteins of His-PYR1, GST-ABI1, His-CPK4 and His-ABF2 were analyzed in 12% SDS-PAGE gel and shown in coomassie staining. (TIF) [file pone.0057171.s004.tif]
