# Supplementary material for: Directly Transforming PCR-Amplified DNA Fragments into Plant Cells Is a Versatile System That Facilitates the Transient Expression Assay
Source: PLoS One. 2013 Feb 26;8(2):e57171. doi: 10.1371/journal.pone.0057171 (PMC3582559; doi:10.1371/journal.pone.0057171)
Supplement: Table S1 — Comparisons of transformation efficiencies in protoplasts. (DOC) [file pone.0057171.s005.doc]

| **Table S1. Comparisons of transformation efficiencies in protoplasts.** | | | | | | |
| --- | --- | --- | --- | --- | --- | --- |
| Repeat | PCR-TES transformation | | | Plasmid transformation | | |
|  | Cells  with  fluore-  scence | Total cells | Efficiency (%) | Cells  with  fluore-  scence | Total cells | Efficiency (%) |
| Repeat 1 | 48 | 85 | 56.5 | 46 | 75 | 61.3 |
|  | 41 | 81 | 50.6 | 66 | 87 | 75.9 |
|  | 53 | 113 | 46.9 | 46 | 65 | 70.8 |
| Repeat 2 | 35 | 54 | 64.8 | 42 | 51 | 82.4 |
|  | 65 | 88 | 73.9 | 42 | 57 | 73.7 |
|  | 49 | 70 | 70.0 | 36 | 46 | 78.3 |
| Repeat 3 | 39 | 61 | 63.9 | 56 | 75 | 74.7 |
|  | 65 | 90 | 72.2 | 37 | 65 | 56.9 |
|  | 58 | 104 | 55.8 | 43 | 54 | 79.6 |
| Repeat 4 | 59 | 110 | 53.6 | 60 | 91 | 65.9 |
|  | 67 | 86 | 77.9 | 75 | 94 | 79.8 |
|  | 35 | 51 | 68.6 | 84 | 104 | 80.8 |
|  | 47 | 72 | 65.3 | 69 | 102 | 67.6 |
| Efficiency (%) | 63.2±2.6 | | | 73.0±2.2 | | |
| Protoplasts were transfected with *p35S-GFP* (Plasmid) or PCR-fragments *35S-GFP-NOS* (PCR-TES) and incubated for overnight. Transformation efficiencies were calculated based on four independent experiments. Data represent the means±SEM. | | | | | | |
