# Supplementary material for: Directly Transforming PCR-Amplified DNA Fragments into Plant Cells Is a Versatile System That Facilitates the Transient Expression Assay
Source: PLoS One. 2013 Feb 26;8(2):e57171. doi: 10.1371/journal.pone.0057171 (PMC3582559; doi:10.1371/journal.pone.0057171)
Supplement: Table S2 — Primer sequences for plasmids constructions and qRT-PCR experiments. (DOC) [file pone.0057171.s006.doc]

| **Table S2. Primer sequences for plasmids constructions and qRT-PCR experiments.** | |
| --- | --- |
| **Name** | **Primer sequence** |
| 35S-MCS | F: 5’-CTCGAGGATCCCGGGAAGAGCGAGCTCGAATTTCCCCGAT-3’  R: 5’-CCGCGGTACCTGCAGAAGAGCTCTAGAGTCCCCCGTGTTCT-3’ |
| Myc | F: 5’-GAGCAAAAGTTGATTTCTGAGGAGGATCTTTAAGAGCTCGAATTT CCCCGAT-3’  R: 5’-GCTCTTCCCGGGATCCTCGA-3’ |
| UBQ10 | F: 5’-CCAAGCTTGCATGCTCAGCTCCACCGTA-3’  R: 5’-TCTTCCATGGGGGCTGTTAATCAGAAAAAC-3’ |
| RD29B | F: 5’-TGCCAAGCTTGTTGAATCTTGCGGAAGCAT-3’  R: 5’-TCTTCCATGGTTCAAGTGAATCAATCAAA-3’ |
| PYR1 | F: 5’-GCAGGTACCGCGGATGCCTTCGGAGTTAACAC-3’  R: 5’-CGGGATCCTCGAGTCACGTCACCTGAGAACCA-3’ |
| ABI1(t-) | F: 5’-CGCCACTAGTATGGAGGAAGTATCTCCGGCGAT -3’  R: 5’-GAGCGGTACCGTTCAAGGGTTTGCTCTT -3’ |
| ABI1 | F: 5’-GCAGGTACCGCGGATGGAGGAAGTATCACCGGCGA-3’  R: 5’-GATCCTCGAGTCAGTTCAAGGGTTTGCTT-3’ |
| SnRK2.6 | F: 5’-GCAGGTACCGCGGATGGATCGACCAGCAGTGA-3’  R: 5’-CGGGATCCTCGAGTCACATTGCGTACACAAT-3’ |
| ABF2 | F: 5’-GCAGGTACCGCGGATGGATGGTAGTATGAATTT-3’  R: 5’-GATCCTCGAGTCACCAAGGTCCCGACTCT-3’ |
| CPKac1 | F: 5’-GCAGGTACCGCGGATGGGTAATACTTGTGTT -3’  R: 5’-CGGGATCCTCGAGCACACCGTCGACTTGAAC -3’ |
| CPKac2 | F: 5’-GCAGGTACCGCGGATGGGTAATGCTTGCGTT -3’  R: 5’-CGGGATCCTCGAGCACACCGTCAATCTGTAC -3’ |
| CPKac3 | F: 5’-GCAGGTACCGCGGATGGGCCACAGACACAGC -3’  R: 5’-CGGGATCCTCGAGCGGTTTGTCTGATGCCTC -3’ |
| CPKac4 | F: 5’-GCAGGTACCGCGGATGGAGAAACCAAACCCT -3’  R: 5’-CGGGATCCTCGAGAGCATGTTCATCAACAAT -3’ |
| CPKac5 | F: 5’-GCAGGTACCGCGGTGTTTCATGGGCAATTCT -3’  R: 5’-CGGGATCCTCGAGAACACCATTCTCACAGAT -3’ |
| CPKac6 | F: 5’-GCAGGTACCGCGGATGGGCAATTCATGTCGT -3’  R: 5’-CGGGATCCTCGAGATCCGGTGCAACTCCATT -3’ |
| CPKac7 | F: 5’-GCAGGTACCGCGGATGGGGAATTGTTGTGGC -3’  R: 5’-CGGGATCCTCGAGCTTCTTTGCATTCAGTAT -3’ |
| CPKac8 | F: 5’-GCAGGTACCGCGGATGGGAAATTGTTGTGCG -3’  R: 5’-CGGGATCCTCGAGCTTCTTCGCATTTTGTAT -3’ |
| CPKac9 | F: 5’-GCAGGTACCGCGGATGGGAAATTGTTTTGCC -3’  R: 5’-CGGGATCCTCGAGTGCTTCTCCACCTTCTCT -3’ |
| CPKac10 | F: 5’-GCAGGTACCGCGGATGGGTAACTGTAACGCCT -3’  R: 5’-CGGGATCCTCGAGTTTCTTTGCATTCTGTAT -3’ |
| CPKac11 | F: 5’-GCAGGTACCGCGGATGGAGACGAAGCCAAAC -3’  R: 5’-CGGGATCCTCGAGTGCTTGTTCATCGACAAT -3’ |
| CPKac12 | F: 5’-GCAGGTACCGCGGATGGCGAACAAACCAAGA -3’  R: 5’-CGGGATCCTCGAGAACCTTATCATCCACAAT -3’ |
| CPKac13 | F: 5’-GCAGGTACCGCGGATGGGAAACTGTTGCAGA -3’  R: 5’-CGGGATCCTCGAGTTTCTTTGCGTTCTGAAT -3’ |
| CPKac15 | F: 5’-GCAGGTACCGCGGATGGGTTGCTTTAGCAGC -3’  R: 5’-CGGGATCCTCGAGTTCTCCTCCTCTGATCCA -3’ |
| CPKac21 | F: 5’-GCAGGTACCGCGGATGGGTTGCTTCAGCAGT -3’  R: 5’-CGGGATCCTCGAGTTCTCCCCCTTTGATCCA -3’ |
| CPKac23 | F: 5’-GCAGGTACCGCGGATGGGTTGTTTCAGCAGT -3’  R: 5’-CGGGATCCTCGAGTTCTCCCCCTTTGATCCA -3’ |
| CPKac26 | F: 5’-GCAGGTACCGCGGATGAAGCACAGCGGTGGG -3’  R: 5’-CGGGATCCTCGAGAACTCCATTTTCACAGAT -3’ |
| CPKac27 | F: 5’-GCAGGTACCGCGGATGGGTTGCTTCAGCAGT -3’  R: 5’-CGGGATCCTCGAGTTCTCCTTCTTTCATCCA -3’ |
| CPKac28 | F: 5’-GCAGGTACCGCGGATGGGTGTCTGTTTCTCC -3’  R: 5’-CGGGATCCTCGAGAGGGATATCAGTAGCATT -3’ |
| CPKac29 | F: 5’-GCAGGTACCGCGGATGCTTCAAAACCAACAT -3’  R: 5’-CGGGATCCTCGAGTTTGGTGTCTGTCATCCAT -3’ |
| CPKac30 | F: 5’-GCAGGTACCGCGGATGGGTAATTGTATCGCCT -3’  R: 5’-CGGGATCCTCGAGTTTCTTTGCATTCTGTAT -3’ |
| CPKac31 | F: 5’-GCAGGTACCGCGGATGGGTTGCTACAGCAGT -3’  R: 5’-CGGGATCCTCGAGTTCTCCGTCTTTCATCCA -3’ |
| CPKac32 | F: 5’-GCAGGTACCGCGGATGGGTAATTGTTGCGGAA -3’  R: 5’-CGGGATCCTCGAGTGTCTTTGCATTCTGTAA -3’ |
| CPKac33 | F: 5’-GCAGGTACCGCGGATGGGGAATTGCTTAGCC -3’  R: 5’-CGGGATCCTCGAGAATAGGCTTATCCGATG -3’ |
| CPK4 | F: 5’-GCAGGTACCGCGGATGGAGAAACCAAACCCT-3’  R: 5’-CGGGATCCTCGAGTTACTTTGGTGAATCAT-3’ |
| YC | F: 5’-GGACTCTAGAATGGACAAGCAGAAGAAC -3’  R:5’-GCGGTACCTGCAGAGCGCCAGCACCAGCACCAGCAC  CCTTGTACAGCTCGTCCAT -3’ |
| YN | F: 5’-GGACTCTAGAATGGTGAGCAAGGGCGAGGA -3’  R: 5’-CGCGGTACCTGCAGGGCCATGATATAGACGTTGT -3’ |
| Fu35S-F  Fu35S-R | F: 5’- CAGCGAGTCAGTGAGCGAGGAA-3’  R: 5’- GCTCTTCCGCGGTACCTGCAGAA-3’ |
| FuNOS-F  FuNOS-R | F: 5’- GCTCTTCTCGAGGATCCCGGGAA-3’  R: 5’- ACTGAGAGTGCACCACGCCATT-3’ |
| FuGFP-F  FuGFP-R | F: 5’- GCAGGTACCGCGGAAGAGCATGGGTAAAGGAGAAGAAC-3’  R: 5’- CGGGATCCTCGAGAAGAGCTTATTTGTATAGTTCATCCA-3’ |
| qRT-PCR  Actin2 | F: 5’- GGTCGTACAACCGGTATT-3’  R: 5’- GGCATGAGGAAGAGAGAA-3’ |
| qRT-PCR  SnRK2.6 | F: 5’-AACTCCTGCTTACATCGCTC-3’  R: 5’-GAAAGGATATGCTCCAACCA-3’ |
| qRT-PCR  RD29B | F: 5’- ACGAGCAAGACCCAGAAGTT-3’  R: 5’- AGGAACAATCTCCTCCGATG-3’ |
| G-SnRK2.6 | F: 5’- GGGTAGAAGGCATTCCGATCTA-3’  R: 5’- GTCTTCAAGATATGCCCCATCT-3’ |
